# Comparative study on the independent and combined effects of omega-3 and vitamin B12 on phospholipids and phospholipase A2 as phospholipid hydrolyzing enzymes in PPA-treated rats as a model for autistic traits

**DOI:** 10.1186/s12944-018-0850-1

**Published:** 2018-08-31

**Authors:** Hanan Alfawaz, Ramesa Shafi Bhat, Manar Al-Mutairi, Osima M. Alnakhli, Abeer Al-Dbass, Mona AlOnazi, Majidh Al-Mrshoud, Iman H. Hasan, Afaf El-Ansary

**Affiliations:** 1 Department of Food Science and Nutrition, College of Food and Agriculture Sciences, King Saud University, P.O. Box 22452, Riyadh, Saudi Arabia; 2 Biochemistry Department, College of Science, King Saud University, Riyadh, 11451 Kingdom of Saudi Arabia; 3 Central Laboratory, Center for Female Scientific and Medical Colleges, King Saud University, Riyadh, Saudi Arabia; 4 Pharmacology and Toxicology Department, College of Pharmacy, Riyadh, Saudi Arabia

**Keywords:** Autism, Propionic acid, Omega-3, Vitamin B12, Phospholipase A2, Phospholipids

## Abstract

**Background:**

Abnormal phospholipid metabolism is a major component of many neurodevelopmental disorders including autism. Oral administration of propionic acid (PPA) can produce behavioral abnormalities and biochemical features in rodents similar to those observed in autism and can thus be used as a model to understand impaired brain fatty acid metabolism in autism.

**Methods:**

The present study was designed to understand alterations in phospholipid metabolism in the brain of a rodent model of autism and to explore omega-3 and vitamin B12 as remedies. Five groups of rats were selected: Group 1 was the control. Group 2 was the rodent model of autism treated with a neurotoxic dose of PPA. Group 3 was given vitamin B12 cobalamin (16.7 mg/kg/day) for 30 days after PPA treatment. Group 4 was given pharmaceutical grade Omega-3 (200 mg cholesterol free-DHA/kg body weight/day), a product of Madre lab, Germany, for 30 days after PPA treatment for 3 days. Group 5 was given a combined dose of ω-3 + Vitamin B12 for the same duration post-PPA treatment. Phospholipid levels and Phospholipase A2 were measured in the brain homogenates of all the groups. ELISA and western blotting were used to detect the cPLA2 protein level.

**Results:**

A significant decrease in phospholipid levels and a significant increase in cPLA2 were found in brain tissue of PPA-treated rats; however, both ω-3 and vitamin B12 were efficient in ameliorating the neurotoxic effect of PPA.

**Conclusion:**

Both ω-3 and vitamin B12 may play a role in ameliorating impaired phospholipid metabolism in autism; however, proper clinical trials are needed.

## Background

Propionic acid (PPA) is a short-chain fatty acid produced in the gut as a metabolite of *Clostridium difficile* species and is higher in feces of autistic patients than of normal controls. El-Ansary et al. [1] designed a rodent model of autism by inducing persistent biochemical autistic features by oral administration of PPA in rat pups, including oxidative stress, neuro-inflammation, altered neurochemistry, and impaired energy and lipid metabolism. Recently, the same model with identical dose and mode of administration of PPA was effective in inducing several behavioral abnormalities, such as social interaction impairment, hyperactivity, and repetitive behaviors, together with histopathological changes such as neuronal loss and astrogliosis [2, 3]. This finding supports the use of PPA in animal models of autism.

Total phospholipids account for 20–25% of brain dry weight and form the major components of bio-membranes. Total phospholipids are critical for brain fluidity and ion permeability as dynamic factors that clearly affect cognition [4]. Dysregulated metabolism characterized by lower levels of plasma and brain phospholipids has been proposed as an etiological mechanism in autism [5]. Moreover, phospholipid fatty acid profiles are thought to be an accurate biomarker for the fatty acid status of the brain [4].

Phospholipid components, such as phosphatidylcholine (PC), phosphatidylethanolamine (PE), phosphatidylethanolamine plasmalogen (PEpl), phosphatidylserine (PS) and phosphoinositides (PI, PIP and PIP2), of the mammalian brain are very important for normal brain development and function. In addition to their function as integral components of membrane structure, PS and PI have unique functions. PI and its phosphorylated derivatives are linked to G-protein coupled phospholipase C (PLC) for the release of inositol trisphosphates (IP3) and diacylglycerol (DG), which in turn serve as second messengers for mobilization of intracellular calcium stores and activation of protein kinase C (PKC), respectively [6–10].

Interestingly, during the third trimester of pregnancy and the first two years of life, the brain undergoes a period of rapid development that is critically affected by nutrient insufficiency. DHA is a nutrient that is required for the proper development of the cognitive, sensory, motor, and perceptual systems during this period [11, 12]. Based on the fact that the neurons are continually forming axons and dendritic extensions with accompanying cell membranes, the growing membrane must be relatively fluid; thus, DHA, as the element producing the most fluidity in cell membranes, is highly recommended as a supplement during this neurodevelopment period. Additionally, the synapses, as the primary functional units of brain circuits, are preferentially made from membranes with adequate DHA [13].

Omega-3 and omega-6 PUFA metabolism occurs through the same pathway as desaturase and elongase enzyme activity. Through this pathway, the essential substrate PUFA n-6 linoleic (LA) and n-3 α-linolenic (ALA) are transformed into n-6 AA and n-3 EPA/DHA, respectively. Through the cyclooxygenase (COX) and lipoxygenase (LOX) pathways, AA and EPA are converted into eicosanoids and lipid mediators. Since PUFA are critically important in brain maturation, rapid conversion of these fatty acids to their metabolites may be implicated in the phenotypic brain abnormalities existing in individuals with autism.

Arachidonic acid (AA) and a number of AA-cascade lipid mediators, presented by prostaglandins (PGs), leukotrienes and other eicosanoids, are well documented to exert a variety of neuromodulatory actions such as wakefulness/sleep regulation, pain sensation, and neuroendocrine regulation [14, 15]. The initial step of the AA (ω-6) signaling cascade is regulated by the action of phospholipase A2 (PLA2) which catalyzes the breakdown of membrane phospholipids, releasing AA. There are three known types of PLA2, Ca^2+^-sensitive, cytosolic PLA2 (cPLA2), the secreted form of PLA2 (sPLA2), and Ca^2+^-insensitive enzyme (iPLA2). Cytosolic PLA2 showed high specificity to AA and catalyzes the release of AA from the *sn-2* position of phospholipids [16]. Cytosolic PLA2 has been shown to play a critical role in neuronal plasticity and glutamate excitotoxicity [17].

Saluja et al. [18] reported that both cPLA2 activity and protein levels are significantly increased during brain ischemia. In astrocyte-neuron cocultures, chronic glutamate toxicity was found to induce the activation of astrocytic PLA2. This process was usually followed by significant increase of AA into the medium and increase of lipoxygenase (LOX) and cyclooxygenase (COX) activities, leading to neuronal death [19]. Interestingly, cPLA2α in mice have lower levels of brain AA, COX-2 mRNA, and protein levels. This finding may suggest that cPLA2 and COX-2 are functionally coupled and that overexpression of these enzymes can be easily related to brain neuroinflammation, previously reported in brain homogenates of rats or hamsters after oral administration of a neurotoxic dose of PPA [1, 20, 21]. Pharmacological studies demonstrate that inhibition of cPLA2 may be promising for the treatment of neuro-inflammation, the etiological mechanism involved in many neurological disorders including autism [22].

Our most recent work showed glutamate excitotoxicity and the impairment of the glutamine-glutamate-GABA circuit as two etiological mechanisms in PPA-induced neurotoxicity [23]. This result motivated our interest in measuring PLA2 protein concentration and phospholipids in brain homogenates of PPA-intoxicated rats and in determining the therapeutic effects of omega-3 and vitamin B12 in ameliorating the neurotoxic effect of PPA.

Currently, western blotting is among the best techniques used to follow the effectiveness of treatments on specific target proteins as it greatly reduces cross-reactions which are a potential drawback of the ELISA technique [24]. In this work, the neurotoxic effects of PPA and the therapeutic effects of B12, ω-3 and the mixture of both on PLA2 phospholipid hydrolyzing enzymes in a rodent model of autism were investigated. In addition, the sensitive and quantitative technique ELISA was used. Additionally, the presence of PLA2 was detected by western blotting.

## Methods

- Animals: A total of 35 young male Western albino rats (80–120 g) were obtained from King Saud University, Riyadh. Rats were randomly allocated to the following groups with seven rats each: Group 1 was given only phosphate buffered saline and considered the control. Group 2 was the oral buffered PPA-treated group treated with the neurotoxic dose of 250 mg/kg body weight/day for 3 days [25]. Group 3 was given oral vitamin B12 with the dose of 16.7 mg cobalamin/kg body weight/day, a product of Dawa Pharmaceutical Co., Limited, Hebei, and China, for 30 days after PPA treatment. Group 4 was given 200 mg cholesterol free-DHA/kg body weight/day, a pharmaceutical grade product of Madre lab, Germany, after PPA treatment for 3 days. Group 5 was given a combined dose of ω − 3 + vitamin B12, for the same duration post-PPA treatment. All groups were kept at a controlled temperature (21 ± 1 °C) with ad libitum access to food and water. All experiments were performed in accordance with national animal care guidelines and were preapproved by the faculty ethics committee, King Saud University.

### Sample collection

At the end of the feeding trials, the animals were killed by decapitation; the brains isolated from sacrificed animals were washed, dissected into small pieces and homogenized in distilled water using a Teflon homogenizer. The homogenate was centrifuged at 650 g for 20 min to remove debris and kept at − 80 °C until further use.

### Measurement of phospholipids

Phospholipid levels were quantified using a phospholipid assay kit from Abnova (Abnova [Taiwan] Corporation) with a linear detection range for the colorimetric assay of 3–200 μM and for the fluorimetric assay of 0.6–20 μM phospholipid.

### Measurement of cPLA2 protein using the ELISA technique

Phospholipase A2 was measured using a kit based on the sandwich ELISA principle, a product of LSBio (Lifespan BioScience, Inc., North America) with a detection range of 3.12–200 ng/ml.

### Measurement of cPLA2 protein using western blotting

To investigate the effect of B12 and ω-3 on cPLA_2_ expression in the brain, we performed western blotting as we recently reported. Briefly, brain samples were homogenized in RIPA buffer containing proteinase inhibitors. Total protein content was assayed using Bradford reagent, and 60 μg protein was separated on SDS-PAGE, electro-transferred onto nitrocellulose membranes, and blocked in 5% skimmed milk in TBS Tween 20. The blocked membranes were probed with rabbit anti-cPLA_2_ and mouse anti-GAPDH primary antibodies (Santa Cruz Biotechnology-454 and Abcam 9482, respectively). After washing, the membranes were incubated with the secondary antibodies and developed using an enhanced chemiluminescence kit (Bio-Rad, USA). The blots were scanned and intensity of the obtained bands was quantified using ImageJ (NIH, USA). The results were normalized to GAPDH and are presented as percent of control.

### Statistical analysis

The results of the present study were expressed as the means ± S.D. All statistical comparisons between the control group and the four studied groups were performed using one-way analysis of variance (ANOVA) tests with Dunnett’s test for multiple comparisons using SPSS (Chicago, IL, USA). Significance was assigned at the level of *p* < 0.05. Receiver operating characteristics (ROC) curve analysis was also performed. The area under the curve (AUC), the degrees of sensitivity and specificity, and cutoff values were calculated.

## Results

**Fig. 1:**
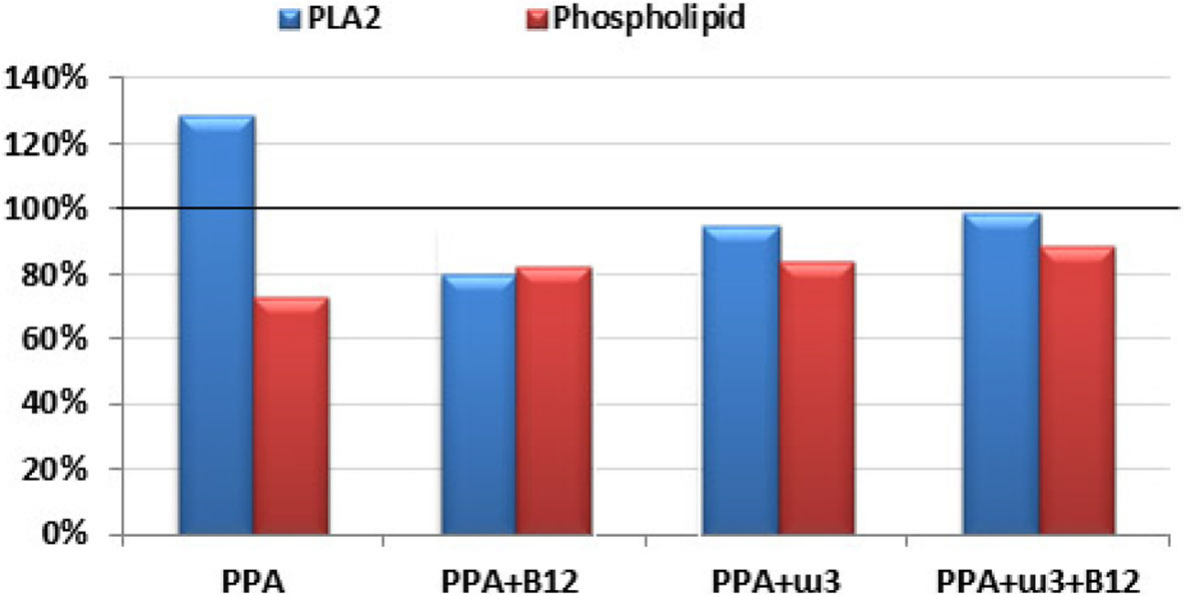
Percentage change of cPLA2 and phospholipids levels in the four studied groups relative to the control group

**Fig. 2:**
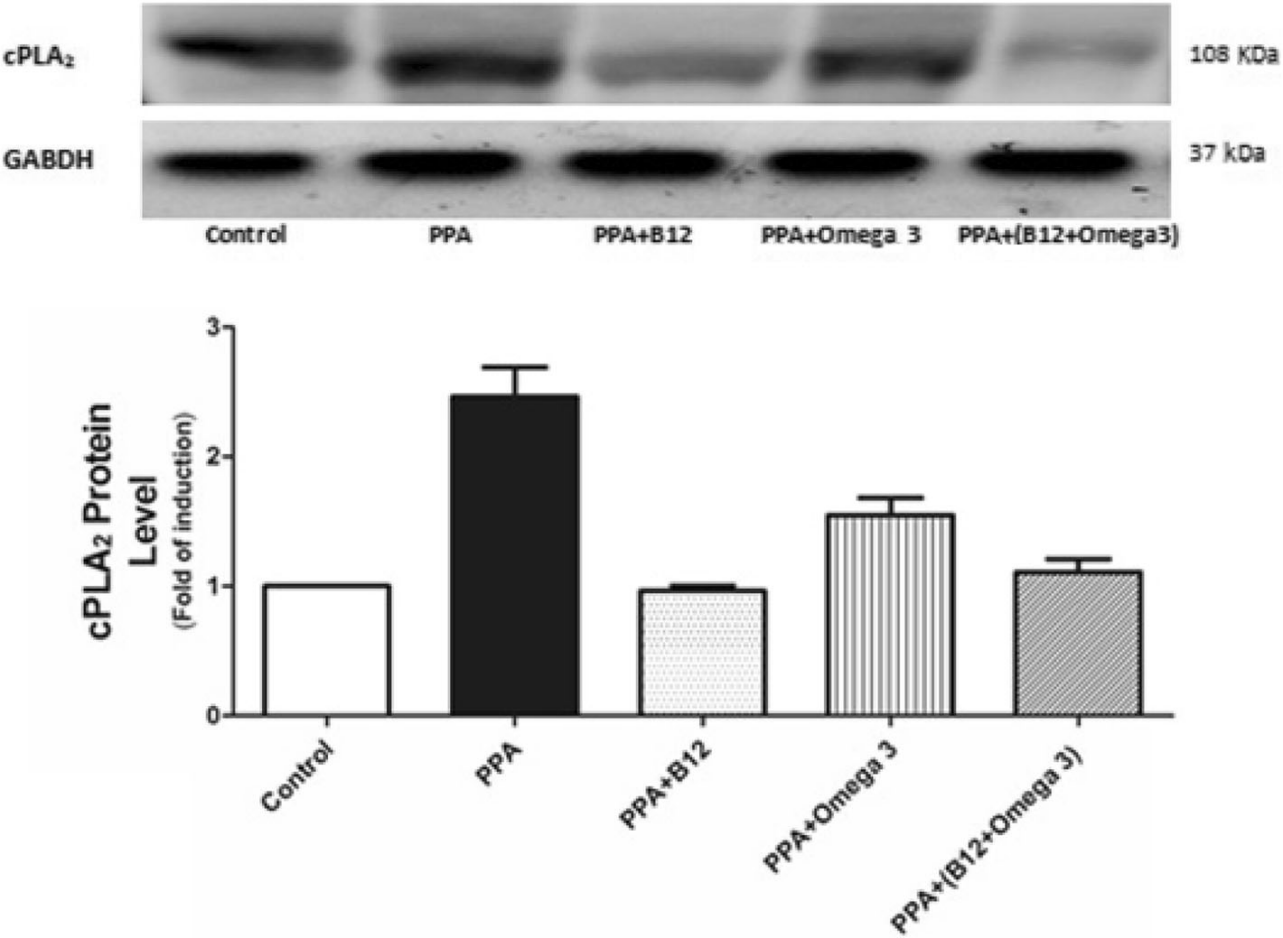
Western blot analysis in whole brain homogenates for cPLA2 in the untreated control, PPA-intoxicated and B12- and ω-3-treated groups

Table 1 and Fig. 1 demonstrate the mean ± S.D. and the percentage change of cPLA2 and phospholipids in the five studied groups. It is clear that PPA induced significant elevation in cPLA2 at 28.54%, together with a significant decrease in phospholipids at 27.14%. Both ω-3 and vitamin B12 were effective in ameliorating the neurotoxic effect of PPA. The three groups treated with ω − 3, vitamin B12, or a combination of both were not significantly different from the control group but were significantly different from the PPA-treated groups. Figure 2 illustrates the overexpression of cPLA2 and the marked attenuation of its expression in PPA–intoxicated and B12- and ω-3-treated groups, respectively.

**Table 1:** Mean ± S.D. of the measured parameters in the five groups studied

Parameter	Group	N	Min.	Max.	Mean ± S.D.	*p* value^**a**^	*p* value^**b**^
PLA2	Control	7	1160.00	2091.90	1441.00 ± 317.85		0.009
	PPA	7	1106.00	2290.10	1852.28 ± 393.77	0.087	
	PPA + B12	7	829.48	1357.13	1153.71 ± 214.77	0.318	
	PPA+ ω 3	7	923.35	1872.70	1364.70 ± 307.00	0.978	
	PPA+ ω-3 + B12	7	853.91	1778.19	1417.81 ± 389.19	1.000	
Phospholipid	Control	7	13.08	21.62	16.76 ± 2.80		0.100
	PPA	7	7.35	16.13	12.21 ± 3.17	0.027	
	PPA + B12	7	10.72	20.18	13.77 ± 3.33	0.211	
	PPA+ ω-3	7	10.48	21.41	14.07 ± 3.48	0.290	
	PPA+ ω-3 + B12	7	12.68	16.79	14.82 ± 1.96	0.566	

^a^*p* value between each group and the control group

^b^*p* value among all groups

Table 2 presents the area under the curve (AUC), together with cutoff values, specificity and sensitivity, for cPLA2 and total phospholipids in PPA-intoxicated rats and those treated with either B12 or ω − 3 independently or in combination.

**Table 2:** ROC-Curve of all parameters in all groups

Parameter	Group	AUC	Cut-off value	Sensitivity %	Specificity%	*p* value
cPLA2	PPA	0.755	1527.550	85.7%	85.7%	0.110
	PPA + B12	0.776	1398.601	100.0%	57.1%	0.085
	PPA+ ω-3	0.571	1403.250	71.4%	57.1%	0.655
	PPA+ ω-3 + B12	0.510	1068.040	28.6%	100.0%	0.949
Phospholipid	PPA	0.878	15.563	85.7%	71.4%	0.018
	PPA + B12	0.816	12.944	71.4%	100.0%	0.048
	PPA+ ω-3	0.816	14.345	85.7%	85.7%	0.048
	PPA+ ω-3 + B12	0.735	16.536	85.7%	57.1%	0.142

## Discussion

In the present study, the significant increase in cPLA2, together with the significant decrease in brain phospholipids, can be easily related to the neurotoxic effect of PPA. The increase in the expression of PLA2 in the PPA-treated group was clearly detected from the WB when compared with the control (Fig. 2). The ameliorating effects of B12, ω-3, and a mixture of both were also clear in the same blot, which confirms the ELISA data obtained and presented in Table 1 and Fig. 1. This result could be related to the effect of vitamin B12 on Δ5 and Δ6 desaturases, two important enzymes critical for the synthesis of PUFA such as EPA, DHA, and AA, in a multistage process of successive catalytic reactions by elongase and desaturase enzymes. Wadhwani et al. [26] proved the effectiveness of vitamin B12 micronutrient supplementation on fatty acid desaturases. Compared with controls, vitamin B12 deficiency during pregnancy is known to reduce Δ6 desaturase activity and mRNA levels of Δ5. Interestingly, the mRNA level of Δ5 desaturase reverts back to normal levels as a result of omega-3 fatty acid supplementation.

The detection of cPLA2 with an average molecular weight of 108 KDa using the WB technique is consistent with the work of Hirashima et al. [27] in which a 110 kDa PLA2 was purified and characterized from bovine brain cytosol. Activation of cPLA2 is usually accompanied by cascade signaling and production of PGE2 as an inflammatory marker. Recognition of the significance of PLA2 in neurodevelopmental inflammatory diseases has made it a very attractive target for drugs. In the present study, B12, ω-3, and a combination of both demonstrate potency in reducing the inflammatory effects of PPA through the attenuation of the increased level of cPLA2 and the decreased concentration of rat brain phospholipids, two neurotoxic features of PPA in the rodent model of autism. This finding is supported by the recent work of Lia et al. [28] in which PLA2 and COX-2 were among the proteins that induced a response to NF-κB activation after subcutaneous injection of aluminum into a mouse as a xenobiotic with immune-stimulating and neurotoxic effects that might be related to the remarkable increase of autism through the routine exposure of infants to aluminum worldwide.

The significant decrease of phospholipids (Table 1) can be related to glutamate excitotoxicity, the neurotoxic mechanism of PPA [24] and to the etiological mechanism repeatedly reported in autistic patients [29]. Based on the most recent finding of Schneider et al. [30], the plasticity related gene 1 (PRG-1) plays a critical role in decreasing presynaptic glutamate release through the interaction with phospholipids. This result was confirmed when deletion of the PRG-1 gene increased glutamate release and increased neuronal glutamate over-excitation. Based on this finding, the significant decrease of phospholipids in the PPA-treated group of this study can be explained by the glutamate excitotoxicity recently reported in juvenile rats with PPA administered orally [24].

Due to the enhanced neurogenesis during brain development early in life, sufficient dietary sources of ω-3 PUFA are needed for maintenance of neuronal functions [31, 32]. This supplement is mostly needed for the synaptic membranes which contain high levels of phospholipids [33]. Findings from animal and clinical studies support the role of ω-3 PUFA as essential nutrients for maintaining normal brain function [34].

Based on the fact that autistic patients and the rodent model of autism demonstrate differences in plasma ω-3 / ω-6 ratios [35–39], ω-3 was suggested as a treatment for autism. The ameliorating effect of ω-3 reported in the present study, shown as a decrease in cPLA2 protein (using ELISA and WB) and the significant increase in the depleted phospholipids, may support the recent interest in the use of this fatty acid as a therapeutic strategy to treat autism. Supplementation with ω-3 for 12 weeks for autistic children ranging from 7 to 18 years old produces significant improvements in all subscales, including blood fatty acid profiles. Unfortunately, once a child already shows the autistic phenotype, ω-3 is unlikely to completely correct the deficits, because there are many other factors involved as etiological mechanisms of this disorder. However, many studies do not exclude the possibility that a subgroup of autistic patients may respond to ω-3 treatment [40]. The remarkable improvement in cPLA2 and phospholipid levels in groups treated with ω-3, vitamin B12, and ω-3 + vitamin B12 is consistent with a recent study by Rathod et al. [41] in which the combined supplementation of vitamin B12 and ω-3 fatty acids led to higher ω-3 and nerve growth factor (NGF) levels in the hippocampus, higher brain derived neurotrophic factor (BDNF) levels in the cortex and hippocampus, together with improvement of cognitive performance. Omega-3 as a precursor for active mediators can regulate various brain functions such as neurotransmission, inflammation, immune reaction and neuronal survival [42–46].

The present study is consistent with the recent work of Qasem et al. [47], which reported a remarkable reduction in phospholipids and an increase in cPLA2 protein levels as etiological mechanisms of autism; these findings might support the use of PPA postnatal exposure to induce persistent autistic features, similar to the mechanism that causes autism. Receiver operating characteristic analysis demonstrated that while cPLA2 can be used as a marker of PPA neurotoxicity (AUC of 0.755, 85.7% specificity, and 85.7% sensitivity), phospholipids can be used to predict both PPA neurotoxicity and the therapeutic potency of B12, ω-3 and their mixture (AUCs of 0.878, 0.816, 0.816, and 0.735 respectively) with satisfactory specificity and sensitivity [48].

## Conclusion

In the present study, it is likely that vitamin B12 and ω-3 fatty acids may act independently or synergistically to ameliorate the neurotoxic effects of impairment of cPLA2 and phospholipids induced in rat pups by orally administered PPA.

## Abbreviations

AA: Arachidonic acid
cPLA2: Cytosolic phospholipase A2
DG: Diacylglycerol
ELISA: Enzyme-linked immunosorbent assay
IP3: Inositol trisphosphates
PC: Phosphatidylcholine
PE: Phosphatidylethanolamine
PEpl: Phosphatidylethanolamine plasmalogen
PGs: Prostaglandins
PKC: Protein kinase C
PLA2: Phospholipase A2
PLC: Phospholipase C
PPA: Propionic acid
PS: Phosphatidylserine
WB: Western blot
